# The Nurse Resilience Scale (NuRSe): Development and Content Validation

**DOI:** 10.1155/jonm/3791713

**Published:** 2026-09-24

**Authors:** Alannah L. Cooper, Desley G. Hegney, Matthew A. Albrecht, Julia Fallon-Ferguson, Janie A. Brown

**Affiliations:** ^1^ School of Nursing, College of Health & Education, Murdoch University, 90 South Street, Murdoch Western Australia, 6150, Australia, murdoch.edu.au; ^2^ School of Nursing and Midwifery, Adelaide University, Frome Road, Adelaide South Australia, 5000, Australia, adelaide.edu.au; ^3^ Western Australian Centre for Road Safety Research, School of Psychological Science, University of Western Australia, Perth, Western Australia, Australia, uwa.edu.au; ^4^ CREST Program, Curtin University, Perth Kent Street, 6102, Bentley, Western Australia, Australia, curtin.edu.au; ^5^ Curtin School of Nursing, Curtin University, Kent Street, Bentley Western Australia, 6102, Australia, curtin.edu.au; ^6^ St John of God Midland Public and Private Hospitals, 1 Clayton Street, Midland Western Australia, 6056, Australia, sjog.org.au; ^7^ JBI Evidence Informed Healthcare Care, Curtin University, Perth Kent Street, 6102, Bentley, Western Australia, Australia, curtin.edu.au

**Keywords:** adversity, Delphi, instrument, measure, nurse, resilience, scale, tool

## Abstract

**Aim:**

To develop and assess the content validity of a profession‐specific measure that incorporates the key attributes known to influence nurse resilience.

**Design:**

Iterative scale development informed by a three‐round expert panel review using an online Delphi technique.

**Methods:**

Scale development was based on the literature and drew on items in existing instruments to capture the reported key attributes of nurse resilience. Content validation of potential items were assessed in the first round, followed by further assessment of closely related items that overlapped or measured the same concept in the second round. In the final round, the first iteration of the nurse resilience scale was presented to the expert panel to assess if the items sufficiently captured the key attributes of nurse resilience.

**Results:**

A 70‐item scale was developed, consisting of 11 domains. The scale comprises 33 items relating to internal factors, 34 items for external factors and three for work‐life balance.

**Conclusion:**

A profession‐specific measure that reflects the complexity of nurse resilience has been developed. The nurse resilience scale demonstrated preliminary content validity, based on the content validity analysis by an expert panel.

**Implications for Nursing Management:**

Following the completion of psychometric testing, the nurse resilience scale can be used by clinical leaders and researchers to assess internal and external factors that influence nurse resilience. This will provide an opportunity to obtain a fuller understanding of the factors that impact nurse resilience and develop optimal interventions to sustain it.

**Reporting Method:**

Conducting and reporting of Delphi studies (CREDES) and guidance on Delphi studies.

## 1. Introduction

Healthcare systems around the world face significant challenges in relation to global nursing shortages that negatively impact the provision of safe patient care [1, 2]. Nursing shortages are worsening as nurses continue to be exposed to unsustainable conditions that are detrimental to retention and recruitment [2]. Key issues adversely affecting nurses highlighted in a recent report from the International Council of Nurses include high workload, inadequate staffing, violence against staff, exposure to suffering and death, working in inefficient systems, poor leadership, poor working conditions and low pay [3].

The challenging work environments nurses are exposed to are reported to have the potential to cause harm. Numerous studies of nurses have identified that they experience higher levels of burnout, depression, anxiety, post‐traumatic stress disorder and higher rates of suicide compared to the general population [4–8]. Poor psychological wellbeing in nurses is associated with negative impacts on patient care [9, 10]. Additionally, higher levels of burnout, stress, anxiety and depression are associated with nurses’ intention to leave the profession [11, 12].

In contrast, higher resilience levels are associated with lower levels of adverse psychological outcomes amongst nurses. In the context of shortages and the increasing challenges faced by nurses in their work, there has been growing attention in practice and research on ways to sustain and maintain nurses in the profession. Promoting nurse resilience may help prevent negative psychological outcomes and sustain nurses in their work [13–15]. With an estimated global shortage of 5.8 million nurses, finding ways to promote nurse resilience in order to increase staff retention and improve patient safety is a high priority [16, 17].

## 2. Background

The concept of nurse resilience began to emerge in the literature in 2012 with the publication of several quantitative research studies [18–20], mirroring the exploration of positive adaptation by individuals in the face of adversity in the discipline of psychology [21]. In the discipline of psychology, resilience was initially considered as an individual trait that was fixed or stable over time [21]. This understanding has evolved to recognise resilience as a complex and dynamic process which varies over context and time and is influenced by internal and external factors [22, 23].

The emerging research examining nurse resilience was followed by a large body of research that investigated the negative psychological outcomes nurses could face as a result of their work, including burnout, stress, compassion fatigue, anxiety and depression [5, 24, 25]. Following the lead of research in psychology, nurse researchers applied pre‐existing measures of resilience, developed outside of a nursing context, to assess for associations and relationships between resilience levels and a wide range of variables including but not limited to age, gender, education level, years of experience, burnout, post‐traumatic stress disorder, depression, anxiety and intention to leave [26, 27]. Examining potential protective factors was an important shift towards considering ways to protect nurses from these known negative psychological outcomes. Researchers adopted general definitions of resilience and instruments (tools or scales) to measure resilience that were developed with non‐nurse samples including patients [22, 28–30], general populations [28, 31], university students [32–34] and employees in nonspecific workplaces [35, 36].

With growing interest and research investigating nurse resilience, members of our research team developed an initial working definition based on the existing literature, which revealed limitations in the understanding of nurse resilience and its key attributes [37]. Most of the research investigating nurse resilience that informed our definition had focused on the individual [13, 14]. This individualistic approach has, however, been widely criticised as it fails to address the complexity of resilience [38, 39]. Due to the underestimation of the influence of external factors and the responsibility organisations have to their employees, we subsequently updated the definition of nurse resilience [38]. The updated definition provides a comprehensive understanding of nurse resilience that considers individual and external factors:

*Resilience is a complex and dynamic process, influenced by individual factors, as well as modifiable workplace conditions, organizational philosophy, management performance, and the teams nurses work within. These factors influence the extent to which resilience can be sustained, to enable nurses to positively adapt to* *workplace stressors, avoid psychological harm, and continue to provide safe, high-quality patient care* ([38], p. 9)

We also extracted the key attributes of nurse resilience from the available literature, which are social support, self‐efficacy, work‐life balance, self‐care, humour, optimism, being realistic, workplace conditions, organisational philosophy, management performance and team factors [38]. In recent years, other researchers have also recognised the importance of considering the influence of external factors on nurse resilience [13, 40, 41]. Several studies have found exposure to worsening workplace conditions can erode nurse resilience confirming the importance of examining these modifiable conditions [42–45].

Having defined nurse resilience, we then assessed the instruments used in studies that have included measures of resilience in samples of nurses. A scoping review was conducted to ascertain how effectively these instruments measure the known attributes of nurse resilience [26]. The review identified that none of the existing instruments, including two profession‐specific instruments [46, 47], reported in the literature captured all the attributes identified in our definition of nurse resilience. Significant limitations with the existing instruments applied to nurses were found, particularly in relation to the lack of consideration of the external factors that influence nurse resilience. For example, none of the instruments included items relating to organisational philosophy or management performance. These deficiencies are reflective of the predominate focus in the literature where resilience has been viewed as an individual issue. The shortcomings of the instruments applied to samples of nurses has resulted in a failure to capture the complexity of nurse resilience. Consequently, the profession is unlikely to have a full understanding of nurse resilience and is therefore unable to develop optimal interventions to sustain nurse resilience. To address this gap, we sought to develop and validate a profession‐specific scale that reflects the definition of nurse resilience and incorporates the key attributes known to influence nurse resilience.

## 3. Methods

### 3.1. Design

A modified online or e‐Delphi technique was used to seek anonymous feedback from expert panel members via a series of Delphi rounds [48, 49]. The study is reported in accordance with the Conducting and Reporting of DElphi studies (CREDES) guidance on Delphi studies [50]. As is accepted in the literature, we used a modified approach to suit our experts and to adapt to their feedback across the rounds, while maintaining the key characteristics of Delphi studies, anonymity, iteration with controlled feedback, statistical group response and input [49]. Through this iterative process, expert panel members refined their opinions to achieve consensus on a profession‐specific scale to measure nurse resilience. There is a recognised gap in defining consensus across Delphi studies [49]; however, for the purposes of this instrument development, the research team considered consensus was reached when high levels of content validity were achieved (all items CVI > 0.78), and experts rated the proposed included constructs as sufficient (> 80%), while addressing the qualitative feedback provided where experts indicated that items should be changed or removed.

**FIGURE 1:**
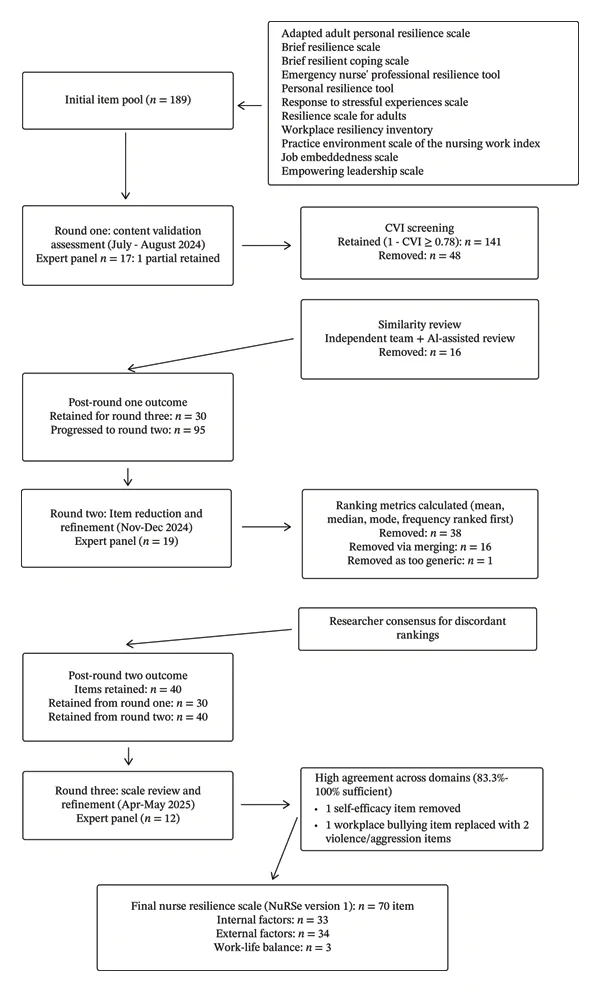
Validation process.

Scale development was based on the literature identified in our earlier scoping review [26] and drew on items from available existing instruments identified to capture the known key attributes of nurse resilience [38], followed by a three‐round expert panel review (Figure 1). The first validation round assessed the content validity of all potential items. In the second‐round, further assessment of closely related items that had demonstrated high levels of content validity was undertaken. In the third and final round, the first iteration of the new draft scale to measure resilience was reviewed by the expert panel. As is the case with consensus, the number of rounds in a Delphi process lacks guidance or consistency in the literature [49]. Our resources, the burden on experts and the content validity results from each round dictated the number of feedback rounds required to reach consensus, which fell within the number (between two and seven) found to be consistently reported in Delphi studies [51].

### 3.2. Instrument

Authors of the existing resilience instruments (*n* = 15) identified in our earlier scoping review [26] were contacted and permission was sought to review instrument items for potential inclusion in the validation process. This resulted in eight instruments being available to review (Table 1) [28, 29, 33, 34, 46, 47, 52, 53]. Four members of the research team independently reviewed the items from the eight resilience instruments against descriptions of the key attributes of nurse resilience: social support, self‐efficacy, work‐life balance, self‐care, humour, optimism, being realistic, workplace conditions, organisational philosophy, management performance and team factors [26].

**TABLE 1:** Key nurse resilience attributes in existing resilience instruments.

Instrument	Social support	Self‐efficacy	Work‐life balance	Self‐care	Humour	Optimism	Being realistic	Workplace conditions	Organisational philosophy	Management performance	Team factors
Adapted Adult personal resilience scale	✗	✔	✗	✗	✗	✗	✗	✗	✗	✗	✗
Brief resilience scale	✗	✗	✗	✗	✗	✗	✗	✗	✗	✗	✗
Brief resilient coping scale	✗	✗	✗	✗	✗	✔	✗	✗	✗	✗	✗
Emergency nurses’ professional resilience tool	✔	✗	✔	✔	✗	✔	✗	✔	✗	✗	✔
Personal resilience tool	✗	✔	✗	✗	✗	✗	✗	✗	✗	✗	✗
Response to stressful experiences scale	✗	✔	✗	✗	✗	✔	✔	✗	✗	✗	✗
Resilience scale for adults	✔	✔	✗	✗	✔	✔	✔	✗	✗	✗	✗
Workplace resiliency inventory	✔	✔	✗	✗	✗	✔	✗	✗	✗	✗	✗

A meeting was then held to discuss the first list of potential items and any adaptations required for the first round of the validation process. Adaptions were made to reflect the Australian context; for example, replacing the term ‘Nursing Administrator’ to ‘Director of Nursing’ to match the title used locally. The researchers also developed new items that drew on our definition of nurse resilience or the key attributes and sought out other instruments that measured attributes that had not been included in existing resilience instruments identified in our earlier scoping review [26]. This included reviewing items, with permission from the instrument developers, from the practice environment scale of the nursing work index [54], the job embeddedness scale [55] and the empowering leadership scale for items that related to key attributes of organisational philosophy and management performance [56]. A total of *n* = 189 potential items to measure the key attributes of nurse resilience were distilled or developed.

The transition from multiple instruments to the 189‐item pool reflects a staged synthesis and consolidation process. Initially, all items extracted from the resilience and related workforce instruments were mapped against the previously defined attributes of nurse resilience [26, 38] and grouped according to attribute to preserve conceptual coverage. Exact duplicate items were removed, while conceptually similar items were clustered to identify overlap without prematurely eliminating potentially important wording. This process also identified gaps in attribute coverage, prompting the development of additional items and review of supplementary instruments. The resulting pool of 189 items represented a comprehensive, de‐duplicated item set for formal content validation by the expert panel.

### 3.3. Sample

To form an expert panel with relevant knowledge and expertise of nurse resilience, invitations were sent to nurses with clinical, academic and management backgrounds, psychologists and members of the International Consortium on Occupational Resilience (https://research.curtin.edu.au/healthsciences/health-sciences-research/research-institutes-centres/icor/). This convenience sample was consistent with recommendations on expert panel selection [57]. While the size of expert panels can vary greatly [58], reviews of the number of experts required indicate a panel should ideally consist of between eight to 23 experts [59].

### 3.4. Expert Panel Administration

The validation process (Figure 1) was conducted online via Qualtrics (Provo, UT). Three members of the research team sent invitations to *n* = 43 potential expert panel members via email for each validation round. All responses from the panel members were anonymous. A short instructional video and written information were provided to participants prior to the first round of validation to outline the purpose of the study, the scope of their involvement, the background to the study and how to complete the validation process. Reminders were sent via email at one and two weeks after the initial distribution of each validation round.

### 3.5. Ethical Considerations and Approvals

Ethical approvals were granted by St John of God Health Care (ref#2025) and Curtin University (HRE2023‐0006) human research ethics committees. Expert panel members were asked to confirm electronically that they had read and understood the written information provided about the study prior to commencing each round. No identifying data were collected from respondents. All data collected were stored electronically and accessible only to members of the research team.

### 3.6. Round One Data Collection and Analysis

A total of *n* = 189 potential items to measure the key attributes of nurse resilience were presented to the expert panel to review the relevancy/representativeness, clarity and comprehensiveness of capturing nurse resilience and its attributes. The definition of nurse resilience and individual definitions of each key attribute were provided to the expert panel to consider as they reviewed each item [38]. There was also the opportunity to provide written comments or feedback for each attribute. Participant responses were included in the analysis if they completed at least two sections (26 items or 24%) of the survey (this was not pre‐specified).

To identify items for further consideration by the expert panel in round two, the initial set of *n* = 189 items were screened for content validity using the content validity index (CVI). Experts were asked to rate the relevancy of each item on a four‐point rating scale (from 1—not relevant to 4—highly relevant). As is typically conducted for CVI metrics [60, 61], responses were dichotomised into ‘relevant’ (if the item scored 3 or above on the relevancy scale) or ‘not relevant’ (if the item scored below 3). Subsequently, for each item, an item‐level CVI (I‐CVI) was calculated as the proportion of all dichotomised ‘relevant’ ratings divided by the total number of responses. The denominator for the I‐CVI calculations included all individuals who responded to that specific question on a question‐by‐question basis.

A modified kappa statistic (*k* ^∗^) [61] was also calculated for each item, representing the agreement beyond chance that the item is relevant. However, *k* ^∗^ values were very similar to all I‐CVI values, adding little additional discriminative ability. Items that received an I‐CVI ≥ 0.78 (effectively ≥ 0.812 due to the number of experts) were accepted as content valid [61, 62] and were considered for the next round of expert evaluation.

All members of the research team independently reviewed the items with a CVI ≥ 0.78 to assess for similarity. In addition to the independent team review, Claude Sonnet (Version 3.5) was used to assess for similarity across items. Outputs were validated through manual inspection of the items, ensuring they were identical to the original items. Similar items as indicated by the large language model were placed next to each other in a spreadsheet by row. The spreadsheet was then used to aid manual review of similar items, where items in the spreadsheet that were co‐localised and indicated as similar enabled more efficient classification by the authors. The research team then met to discuss each team member’s review and the areas of similarity detected via the large language model to reach an agreement on which items could be removed, retained and/or required further review by the expert panel in round two. Open‐ended responses received for each attribute were considered by the research team and minor revisions to items were made where appropriate.

### 3.7. Round One Results

Seventeen experts anonymously submitted the content validity assessment in the first administration conducted between July–August 2024, representing a 40% response rate. One of those experts partially completed the validation round (∼33% completed). The partially completed assessment was kept for analysis of the completed sections. The level of agreement of content validity by the expert panel was high with *n* = 141 items reaching the critical threshold criteria of 0.78 for the I‐CVI and *k* ^∗^ (Supporting File 1). The *n* = 48 items with an I‐CVI and *k* ^∗^ < 0.78 were removed. Following the review for similarity across the items with an I‐CVI and *k* ^∗^ ≥ 0.78, a further *n* = 16 items were removed. Thirty items were retained and did not require further input from the expert panel (reserved for round three), leaving *n* = 95 items for further review by the expert panel in the second validation round.

### 3.8. Round Two Data Collection and Analysis

Further assessment was sought on closely related items that overlapped or measured the same concept and had high content validity scores in the first administration. The expert panel was asked to rank *n* = 55 items that were identified as measuring the same concept (out of 20 identified concepts) and had an opportunity to provide open‐ended feedback for each ranking question. The mean, median and mode of the rankings, along with the number of times the item was ranked first were calculated for each item. Items that won on all dimensions were selected to progress for further review. For items where there was disagreement among the metrics, the researchers discussed which of the highest‐ranking items should progress. To do this, a dedicated item inclusion/exclusion meeting was held where all researchers discussed each conflicting item ranking set sequentially. Following the discussion of each, all researchers were required to agree with that item’s inclusion for an item to be preferred.

Feedback was sought to clarify or re‐word items, including the potential to merge some items that related to either different healthcare team members or levels of healthcare management. Experts were asked whether *n* = 16 items could be merged within the group of similar items, with the response being ‘yes’ or ‘no’. If ≥ 75% of experts thought the items could be merged, the team evaluated the possibility of merging alongside the experts’ comments. Suggested rewording of items via open‐ended responses was collated by one member of the research team before independent review by all members of the research team. Comments were organised according to the questionnaire item to which they referred and examined for suggestions relating to wording, clarity, relevance, duplication or missing concepts. Proposed amendments were discussed by the research team and implemented only where agreement was reached.

### 3.9. Round Two Results

Further assessment of items that overlapped or measured the same concept and had achieved high levels of content validity in the first administration was completed by *n* = 19 experts (44% response rate) between November and December 2024. The further review of the *n* = 95 items by the expert panel led to the exclusion of *n* = 38 items based on selecting the top ranked item for sets of items that were identified as covering similar content. Feedback on the potential to merge similar items led to a further reduction of *n* = 16 items. The consolidation of the retained items from round one and two also led to the removal of an additional item with feedback from the expert panel that the item was too generic. This left *n* = 40 items from the second round of validation which were then combined with the *n* = 30 items retained from round one resulting in *n* = 70 items.

Given the importance of considering both internal and external factors to comprehensively assess nurse resilience, the research team then examined the number of items per attribute. A total of *n* = 34 items were included in the attributes that captured internal factors, namely, being realistic, humour, optimism, self‐care, self‐efficacy and social support. There were *n* = 33 items included in the attributes related to external factors, management performance, organisational philosophy, team factors and workplace conditions. The attribute of work‐life balance was considered to crossover internal and external factors and consisted of *n* = 3 items.

### 3.10. Round Three Data Collection and Analysis

The first iteration of the NuRSe was presented to the expert panel for review. The proposed items for each attribute were listed alongside the definition of the attribute, for the panel to consider and answer the following questions:

• Do the items sufficiently capture the attribute?
• Are there any items you would change?
• Are any items for this attribute redundant/could be removed?

These questions had options of ‘yes’, ‘no’, or ‘unsure’, and panel members then had the opportunity to provide open‐ended feedback to elaborate on their responses. After considering all the attributes of nurse resilience, respondents were asked to provide their opinion on whether the weighting of each attribute in the scale was appropriate (open‐ended response) and were also asked what the most appropriate timeframe would be to ask nurses to focus on when completing the scale (in the last month/in the last week/others). The round concluded with an opportunity to provide any final comments on the scale. The proportion of experts indicating that each domain was sufficient, needed change or could have items removed was calculated. Again, all qualitative feedback was collated and considered and discussed amongst the researchers and changes made where appropriate. Given that the experts considered most constructs sufficient and that there were low proportions of experts indicating ‘change’ or ‘remove’, this step relied more heavily on the qualitative feedback provided to adjust or remove questionnaire items within the instrument.

### 3.11. Round Three Results

The first iteration of the NuRSe was reviewed by *n* = 12 experts (28% response rate) between April and May 2025. There were high levels of agreement that the items included in the first iteration of NuRSE sufficiently captured each of the key attributes of nurse resilience (Table 2). Open‐ended responses received for each attribute were again considered by the research team and minor revisions to items were made where appropriate. One item was removed for the attribute of self‐efficacy based on feedback from the expert panel that the item was vague and redundant.

**TABLE 2:** Proportion of experts rating the questions in each domain as ‘sufficient’.

Domain	Total responses	Sufficient			
		Yes	No	Unsure	% yes
Being realistic	14	14	0	0	100
Humour	13	12	0	1	92.3
Management performance	13	13	0	0	100
Optimism	12	11	0	1	91.7
Organisational philosophy	12	12	0	0	100
Self‐care	12	10	1	1	83.3
Self‐efficacy	12	12	0	0	100
Social support	12	12	0	0	100
Team factors	12	10	1	1	83.3
Work‐life balance	12	10	2	0	83.3
Workplace conditions	12	10	2	0	83.3

Specific participant feedback was received that the item for the attribute Workplace Conditions, ‘bullying is not tolerated in my workplace’, did not sufficiently capture the types and sources of violence and aggression nurses are exposed to. The research team used this feedback to split the original item into *n* = 2 new items, with one focussing specifically on workplace violence and aggression from patients and visitors and one focussing on workplace violence and aggression from staff members. Given that the feedback provided on this item directed this action, the *n* = 2 new items were not returned to the expert panel. The removal of two items followed by the addition of two new items resulted in the first version of NuRSe comprising of *n* = 70 items. These refinements resulted in *n* = 33 items for attributes relating to internal factors, *n* = 34 items for external factor attributes and *n* = 3 for work‐life balance. The final items for each attribute are presented in Table 3.

**TABLE 3:** Final items per attribute.

Attribute	Items
Being realistic	(1) It is okay that not every day at work will be a good day (2) I maintain a realistic perspective on what I can achieve (3) I understand that my patients will not always have positive outcomes (4) During and after life’s most stressful events I tend to put things in perspective and realise I will have times of joy and times of sadness (5) During and after life’s most stressful events I tend to be good at determining what situations are changeable and what situations are not

Humour	(1) I can usually see the funny side of things (2) I use humour to relieve tension (3) I use humour in my interactions with patients (4) I laugh with my nursing colleagues (5) I laugh with my multidisciplinary team colleagues

Optimism	(1) I am optimistic about my work (2) I am optimistic about the future of health‐care provision (3) During and after life’s most stressful events, I tend to find the meaning, purpose or mission of my life (4) I have a positive outlook (5) I see challenges as an opportunity for growth (6) There is an optimistic feeling among my nursing colleagues about the work we do (7) There is an optimistic feeling among my multidisciplinary team colleagues about the work we do

Self‐care	(1) I actively take care of my physical health (2) I actively take care of my mental health (3) I prioritise getting a good night’s sleep (4) I spend time with my friends, family, and colleagues

Self‐efficacy	(1) I am confident in my abilities as a nurse (2) I work hard to reach my goals (3) When faced with a difficult situation, I find ways to solve it (4) I apply coping strategies in difficult situations (5) During and after life’s most stressful events, I tend to practice ways to handle it better next time (6) I believe I have the competence to do my job well

Social support	(1) I know that someone will make time to provide me with support if I need them (2) I have people I can rely on in my personal life (3) I have people at work I feel safe debriefing with (4) I know where to seek support in my workplace (5) I receive support at work from my nursing colleagues (6) I receive support at work from my multidisciplinary colleagues

Management performance	(1) My nurse manager empowers me in my work (2) My nurse manager makes me feel valued (3) My nurse manager communicates well with the team (4) My nurse manager uses mistakes as learning opportunities, not criticism (5) My nurse manager is understanding when I am having difficulties (6) My nurse manager makes me feel supported (7) My nurse manager is a good leader (8) My nurse manager addresses my concerns (9) The director of nursing (or equivalent) is a good leader (10) The organisation’s executive team are good leaders (11) The organisation’s executive team listens and responds to employee concerns (12) The organisation’s executive team expects high standards of nursing care (13) In this organisation, nurses who provide direct patient care have a say in how care is planned, delivered and provided

Organisational philosophy	(1) I feel that the organisation I work for values me (2) I feel that the organisation I work for values nurses (3) I feel that the organisation I work for upholds the values it promotes (4) I feel like I am a good match for this organisation (5) My values align with the organisation’s philosophy

Team factors	(1) I work with a good team (2) I work closely with other members of the care team during a crisis or medical emergencies (3) I work in a place that has a positive culture (4) Teamwork is supported and promoted in my workplace (5) I feel respected by my nursing colleagues (6) Medical colleagues listen to concerns

Workplace conditions	(1) I have a say in the conditions of my job (2) My work is recognised in meaningful ways (3) I am paid fairly for the work I do (4) The work I do is within my scope of practice (5) Workplace violence and aggression from patients and visitors is not tolerated in my workplace (6) Workplace violence and aggression from staff members are not tolerated in my workplace (7) My nursing team has an adequate skill mix (8) The multidisciplinary team I work within has the skills needed to meet patient care needs (9) Patient care where I work is of high quality (10) There are sufficient resources (e.g. staffing, equipment and time) to do my job effectively

Work‐life balance	(1) I have enough time to look after my home/family/self (2) I am able to take my leave at a time that suits me (3) I am able to enjoy my time away from work

Open‐ended responses indicated that members of the expert panel thought the scale was appropriately weighted. Most of the expert panel 77% (*n* = 10) thought that the most appropriate timeframe to ask nurses to think about when completing the scale was in the last month. To reflect the 1‐month timeframe that participants will consider when completing NuRSe, a five‐point Likert Scale ranging from ‘not true at all’ (1) to ‘true all of the time’ (5) was selected by the research team, balancing the pros and cons of an instrument with higher sensitivity/better psychometric properties versus speed, simplicity and preference for participants. Previous research has shown psychometric advantages with an increasing number of anchors noting a plateauing effect beginning around 6–7 anchors [63]. Balancing the psychometric properties is the consideration that users perceive Likert scales with fewer anchors as faster to complete and easier to use [64]. The research team subsequently decided that five anchors balanced the psychometric properties with participant experience. The scale has a total possible score of 350 with higher scores reflecting higher levels of resilience. The scale facilitates the assessment of overall resilience through the total score, and the domain scores can be used to identify strengths and deficiencies across the key attributes of nurse resilience.

## 4. Discussion

The aim of this study was to develop and validate a profession‐specific scale that incorporates the key attributes known to influence nurse resilience. Following three rounds of expert consultation, a 70‐item scale was developed reflecting the 11 attributes of nurse resilience [38]. The final distribution of retained items across attributes provides empirical support for the conceptualisation of nurse resilience as influenced by both internal and external (organisational) determinants. Expert‐driven item retention resulted in comparable representation of internal attributes (e.g., self‐efficacy, optimism and self‐care) and external attributes (e.g., workplace conditions, organisational philosophy, management performance and team factors), with a near‐equal allocation across domains. Panel consensus independently supported the importance of both individual and organisational influences on resilience. Experts rated items on the scale as possessing high content validity and the items within each domain as generally sufficient to capture the attributes of nurse resilience, endorsing the comprehensiveness of both individual‐ and system‐level constructs. Importantly, no systematic bias in ratings was observed favouring either internal or external domains, suggesting expert agreement that both domains were essential rather than competing explanations of resilience. Therefore, the NuRSe is unique within the occupation‐specific resilience field in that it possesses attributes across both internal and external factors and includes all key attributes of nurse resilience.

The development of the NuRSe addresses the gaps identified in existing measures of resilience and captures the complexity of nurse resilience [26]. Importantly this includes the external factors that influence nurse resilience that have had limited consideration in research [13, 40, 41]. This is especially important in the context of emerging evidence that worsening work conditions can erode nurse resilience [42–45]. When resilience is measured only at the individual level, nurses’ distress may be interpreted as a deficit in coping capacity rather than a reasonable response to sustained exposure to factors such as understaffing, moral distress, poor leadership or workplace violence [13, 40, 41]. This framing can inadvertently pathologise nurses, obscure organisational contributors to distress and perpetuate inequities by shifting responsibility away from healthcare organisations and leaders. As a result, interventions are frequently directed at individual behaviour change rather than addressing modifiable workplace conditions.

The NuRSe addresses this limitation by explicitly measuring both individual and organisational determinants of resilience, thereby supporting a more equitable and accountable approach. By capturing management performance, organisational philosophy, team factors, and workplace conditions alongside individual attributes, NuRSe enables differentiation between personal adaptive capacity and contextual constraints. This makes structural contributors to reduced resilience visible and measurable, facilitating organisational accountability and system‐level intervention. In doing so, NuRSe reframes resilience as a shared responsibility between nurses and the systems in which they work, aligning measurement with ethical workforce practices and supporting fairer, more effective responses to nurse wellbeing and retention challenges. The final domain structure directly reflects patterns observed during expert review items presenting external influences such as organisational philosophy, management performance and workplace conditions demonstrated strong relevance ratings and were retained throughout the validation process. Where item reduction occurred within these domains, they were primarily due to conceptual overlap between items rather than disagreement regarding their importance of organisational constructs. For example, in round two, refinement resulted in the consolidation of management and team‐related items, indicating conceptual density and expert recognition of multiple overlapping organisational influences on resilience.

While it is critical to avoid individualising systemic problems, individual factors remain an essential component of nurse resilience [37, 38]. Nurses bring individual attributes that shape day‐to‐day functioning, recovery from adversity and engagement with support strategies, and these are particularly relevant for education, professional development and early‐career support. Excluding individual factors from resilience measurement risks oversimplifying resilience as purely structural and fails to acknowledge nurses’ agency, strengths and adaptive capacities [26]. The NuRSe addresses this complexity by deliberately integrating individual attributes alongside organisational and environmental factors, rather than positioning them in opposition. This balance is also empirically reflected in the item development process. During expert review, both internal and external attributes were retained at comparable rates, and item reduction patterns did not preferentially eliminate either internal or external attributes, with no evidence that either domain was systematically prioritised or discounted by the expert panel. Instead, attrition occurred largely through the removal of redundancy and refinement of wording clarity across both, suggesting that experts did not view organisational factors as peripheral, but as an integral and conceptually overlapping component of nurse resilience.

This balanced approach recognises that resilience is co‐produced: individual capacities influence how nurses navigate adversity, while organisational contexts determine the extent to which those capacities can be sustained or eroded. By measuring both domains, NuRSe allows for nuanced interpretation of resilience scores, supports targeted individual and system‐level interventions and avoids both victim‐blaming and organisational absolution. In doing so, the scale provides an ethically defensible and practically useful framework for understanding, supporting and strengthening nurse resilience. This integrated conceptualisation is empirically grounded in the structure of the final NuRSe, where expert consensus across three rounds produced a stable 70‐item instrument with near‐equivalent representation of internal and external factors. There was convergence of high content validity ratings, systematic item retention across both domains and expert‐driven consolidation of overlapping constructs, supporting for the framing of resilience as influenced by internal and external factors.

The NuRSe may provide a structured mechanism for translating resilience data into actionable workforce and management strategies. By disaggregating resilience into individual and organisational domains, the scale enables nurse leaders and health service managers to identify specific points for intervention. For example, low scores in organisational philosophy or management performance domains may indicate the need for leadership development or organisation‐wide interventions, whereas lower scores in individual resilience attributes may indicate the need for targeted professional development, mentoring or transition‐to‐practice support for early career nurses. At a workforce planning level, aggregated NuRSe data can be used to identify units or services at risk of lower resilience, informing proactive allocation of resources, staffing adjustments, or targeted interventions.

Importantly, the scale also enables the evaluation of intervention effectiveness over time. Changes in domain‐specific scores can be used to assess whether interventions are producing measurable improvements in resilience. This supports more precise monitoring of workforce resilience and more accountable investment in resilience initiatives.

While the next stage of development will involve large‐sample pilot testing to establish test–retest reliability, internal consistency, and construct validity through factor analysis, this step is also critical for confirming the utility of domain‐level scoring for applied decision‐making. Establishing psychometric robustness will ensure that NuRSe can be confidently used not only as a research instrument but also as a practical tool to guide workforce planning, targeted intervention design and organisational accountability for nurse wellbeing outcomes.

## 5. Limitations

The content validation of NuRSe was undertaken with a predominately Australian expert panel via a convenience sample which may limit the generalisability of the instrument to other nursing populations. We recommend content validity determination in other contexts prior to application [65]. Further, the study relied on an initial item generation and consolidation process followed by expert judgement as the primary basis for item retention and reduction, which may introduce subjective bias despite structured CVI procedures and consensus processes. While multiple rounds and accepted processes were used to reduce bias in item selection and consolidation, some degree of interpretive influence in refining and merging items is inherent in expert‐driven scale development. Psychometric properties beyond content validity, including construct validity, test–retest reliability and factor structure, are not yet established.

## 6. Conclusion

The NuRSe has been developed based on the literature and drew on items from available existing instruments to capture the 11 key attributes of nurse resilience. This resulted in an instrument that includes internal and external factors and reflects the complexity of nurse resilience. The NuRSe has undergone content validity and will be subjected to further validation, when it is administered in future pilot testing with large samples of nurses. Given the deficiencies in pre‐existing instruments used to measure resilience in samples of nurses, it is anticipated that this profession‐specific instrument will assist clinical leaders and researchers to more effectively assess nurse resilience and develop optimal interventions to sustain it.

## Author Contributions

Conceptualisation: Alannah L. Cooper, Desley G. Hegney, Matthew A. Albrecht and Janie A. Brown. Methodology: Alannah L. Cooper, Desley G. Hegney, Matthew A. Albrecht and Janie A. Brown. Formal analysis: Matthew A. Albrecht, Alannah L. Cooper, Desley G. Hegney, Julia Fallon‐Ferguson and Janie A. Brown. Investigation: Alannah L. Cooper, Desley G. Hegney and Janie A. Brown. Writing–Original Draft: Alannah L. Cooper. Writing–Review and Editing: Desley G. Hegney, Matthew A. Albrecht, Julia Fallon‐Ferguson and Janie A. Brown. Funding acquisition: Alannah L. Cooper, Desley G. Hegney, Matthew A. Albrecht and Janie A. Brown.

## Funding

The completion of this research was supported as part of an AUD$15,000 Early Career Researcher Grant awarded to AC from the St John of God Foundation. Open access publishing was facilitated by Murdoch University, as part of the Wiley–Murdoch University agreement via the Council of Australasian University Librarians.

## Conflicts of Interest

The authors declare no conflicts of interest.

## Supporting Information

Additional supporting information can be found online in the Supporting Information section.

## Supporting information


**Supporting Information** Supporting File 1: a table with the round one content validity results per item, including I‐CVI and *k* ^∗^ values.

## Data Availability

The data that support the findings of this study are available from the corresponding author upon reasonable request.
