# Supplementary material for: The Nurse Resilience Scale (NuRSe): Development and Content Validation
Source: J Nurs Manag. 2026 Sep 24;2026:3791713. doi: 10.1155/jonm/3791713 (PMC13612967; doi:10.1155/jonm/3791713)
Supplement: Supplementary file 1 [file JONM-2026-3791713-s001.docx]

|  | **Question** | **Total Agree** | **Total Respond** | **CVI** | **k*** | **CVI > 0. 78** |
| --- | --- | --- | --- | --- | --- | --- |
| ***Social*** | |  |  |  |  |  |
|  | 1. I have some close friends/family members who value my abilities | 13 | 17 | 0.77 | 0.76 | FALSE |
|  | 2. I know there is someone I can count on to be there for me | 17 | 17 | 1.00 | 1.00 | TRUE |
|  | 3. I have some close friends/family members who are good at encouraging me | 15 | 17 | 0.88 | 0.88 | TRUE |
|  | 4. I know that someone will make time for me if I need them | 17 | 17 | 1.00 | 1.00 | TRUE |
|  | 5. I have people at work I feel safe debriefing with | 17 | 17 | 1.00 | 1.00 | TRUE |
|  | 6. I can discuss personal matters with friends/family members | 15 | 17 | 0.88 | 0.88 | TRUE |
|  | 7. My nursing colleagues are a source of support | 17 | 17 | 1.00 | 1.00 | TRUE |
|  | 8. I easily establish new friendships | 6 | 17 | 0.35 | 0.29 | FALSE |
|  | 9. My multidisciplinary team colleagues are a source of support | 14 | 17 | 0.82 | 0.82 | TRUE |
|  | 10. I enjoy being with other people | 9 | 17 | 0.53 | 0.42 | FALSE |
|  | 11. I have people I can rely on in my personal life | 16 | 17 | 0.94 | 0.94 | TRUE |
|  | 12. I enjoy spending time with friends and family | 12 | 17 | 0.71 | 0.69 | FALSE |
|  | 13. I always have someone who can help me when needed | 16 | 17 | 0.94 | 0.94 | TRUE |
|  | 14. I feel that there is somebody I can talk to that will listen to my problems and concerns | 17 | 17 | 1.00 | 1.00 | TRUE |
|  | 15. I know where to seek support in my workplace | 17 | 17 | 1.00 | 1.00 | TRUE |
|  | 16. My family and friends understand my work situation and feelings | 13 | 17 | 0.77 | 0.76 | FALSE |
|  | 17. I know where to seek help when I need it | 16 | 17 | 0.94 | 0.94 | TRUE |
|  | 18. I have some close friends/family members who back me up | 16 | 17 | 0.94 | 0.94 | TRUE |
|  | 19. There are strong bonds in my family | 9 | 17 | 0.53 | 0.42 | FALSE |
|  | 20. I spend time with my friends, family and colleagues | 13 | 17 | 0.77 | 0.76 | FALSE |
|  |  |  |  |  |  |  |
| ***Self-Efficacy*** | |  |  |  |  |  |
|  | 1. During and after life's most stressful events I tend to find a way to do what’s necessary to carry on | 16 | 17 | 0.94 | 0.94 | TRUE |
|  | 2. I believe that I have the knowledge to do my job well | 15 | 17 | 0.88 | 0.88 | TRUE |
|  | 3. No matter what happens I always find a solution | 15 | 17 | 0.88 | 0.88 | TRUE |
|  | 4. I believe that I am capable of doing my job | 16 | 17 | 0.94 | 0.94 | TRUE |
|  | 5. I will re-establish myself even if the odds are against me | 14 | 17 | 0.82 | 0.82 | TRUE |
|  | 6. During and after life's most stressful events I tend to expect that I can handle it | 12 | 17 | 0.71 | 0.69 | FALSE |
|  | 7. I know that I can solve my personal problems | 12 | 17 | 0.71 | 0.69 | FALSE |
|  | 8. I am able to cope with difficult situations | 15 | 17 | 0.88 | 0.88 | TRUE |
|  | 9. I persevere when faced with adversity | 14 | 17 | 0.82 | 0.82 | TRUE |
|  | 10. I believe that I have the skills to do my job well | 16 | 17 | 0.94 | 0.94 | TRUE |
|  | 11. I keep trying for the things I want until I reach them | 13 | 17 | 0.77 | 0.76 | FALSE |
|  | 12. I am confident in my abilities as a nurse | 14 | 17 | 0.82 | 0.82 | TRUE |
|  | 13. I believe that I can control the situation so I can achieve my daily goals (during a shift) | 12 | 17 | 0.71 | 0.69 | FALSE |
|  | 14. I know that I will succeed if I carry on | 13 | 17 | 0.77 | 0.76 | FALSE |
|  | 15. I will stay with an objective until I have achieved it | 11 | 17 | 0.65 | 0.61 | FALSE |
|  | 16. I am able to bounce back from any kind of adversity | 14 | 17 | 0.82 | 0.82 | TRUE |
|  | 17. I believe that I have the attitude to do my job well | 16 | 17 | 0.94 | 0.94 | TRUE |
|  | 18. I work hard to reach my goals | 14 | 17 | 0.82 | 0.82 | TRUE |
|  | 19. I can recover from just about any type of misfortune | 7 | 17 | 0.41 | 0.31 | FALSE |
|  | 20. When faced with a difficult situation, I find ways to solve it | 15 | 17 | 0.88 | 0.88 | TRUE |
|  | 21. I push myself to succeed | 11 | 17 | 0.65 | 0.61 | FALSE |
|  | 22. I know how to reach my goals | 12 | 17 | 0.71 | 0.69 | FALSE |
|  | 23. I will not let things stop me from achieving my goals | 8 | 17 | 0.47 | 0.35 | FALSE |
|  | 24. I believe that I am a “good” nurse | 13 | 17 | 0.77 | 0.76 | FALSE |
|  | 25. I will regain myself after almost any kind of setback | 11 | 17 | 0.65 | 0.61 | FALSE |
|  | 26. I don’t give up easily | 12 | 17 | 0.71 | 0.69 | FALSE |
|  |  |  |  |  |  |  |
| ***Work Life Balance*** | |  |  |  |  |  |
|  | 1. I travel to reduce the stress of my workplace problems | 4 | 17 | 0.24 | 0.22 | FALSE |
|  | 2. I am not required to do overtime | 9 | 17 | 0.53 | 0.42 | FALSE |
|  | 3. I make time for hobbies and interests outside of work | 15 | 17 | 0.88 | 0.88 | TRUE |
|  | 4. I do not bring the problems of my workplace into the normal course of my life | 13 | 17 | 0.77 | 0.76 | FALSE |
|  | 5. I am able to enjoy my time away from work | 17 | 17 | 1.00 | 1.00 | TRUE |
|  | 6. I do not work outside of the hours I am paid | 12 | 17 | 0.71 | 0.69 | FALSE |
|  | 7. I am able to take my leave at a time that suits me | 14 | 17 | 0.82 | 0.82 | TRUE |
|  | 8. I have enough time to look after my home/family/self | 15 | 17 | 0.88 | 0.88 | TRUE |
|  | 9. I leave work related stress at work | 12 | 17 | 0.71 | 0.69 | FALSE |
|  | 10. I spend time with my friends, family and colleagues | 14 | 17 | 0.82 | 0.82 | TRUE |
|  | 11. I am able to utilise my leave entitlements | 14 | 17 | 0.82 | 0.82 | TRUE |
|  | 12. I have personal plans and interests and I follow them. (e. g. sports, art activities, music, relaxation, and meditation techniques, study) | 17 | 17 | 1.00 | 1.00 | TRUE |
|  | 13. I am able to have a say in my roster | 16 | 17 | 0.94 | 0.94 | TRUE |
|  | 14. I have a good work-life balance | 16 | 17 | 0.94 | 0.94 | TRUE |
|  | 15. I am able to enjoy non-work time without thinking about work | 15 | 17 | 0.88 | 0.88 | TRUE |
|  |  |  |  |  |  |  |
| ***Self-Care Practices*** | |  |  |  |  |  |
|  | 1. I prioritise getting a good night sleep | 16 | 16 | 1.00 | 1.00 | TRUE |
|  | 2. I spend time with my friends, family and colleagues | 15 | 16 | 0.94 | 0.94 | TRUE |
|  | 3. I actively take care of my spiritual health | 8 | 16 | 0.50 | 0.38 | FALSE |
|  | 4. I exercise regularly | 13 | 16 | 0.81 | 0.81 | TRUE |
|  | 5. I travel to reduce the stress of my workplace problems | 5 | 16 | 0.31 | 0.26 | FALSE |
|  | 6. I have a healthy diet | 15 | 16 | 0.94 | 0.94 | TRUE |
|  | 7. I actively take care of my physical health | 16 | 16 | 1.00 | 1.00 | TRUE |
|  | 8. I take time to relax outside of work | 16 | 16 | 1.00 | 1.00 | TRUE |
|  | 9. I have personal plans and interests and I follow them. (e. g. sports, art activities, music, relaxation, and meditation techniques, study) | 14 | 16 | 0.88 | 0.88 | TRUE |
|  | 10. I feel healthy | 11 | 16 | 0.69 | 0.67 | FALSE |
|  | 11. I actively take care of my mental health | 15 | 16 | 0.94 | 0.94 | TRUE |
|  |  |  |  |  |  |  |
| ***Humour*** | |  |  |  |  |  |
|  | 1. I laugh with my patients and their families | 15 | 16 | 0.94 | 0.94 | TRUE |
|  | 2. I laugh with my multidisciplinary team colleagues | 16 | 16 | 1.00 | 1.00 | TRUE |
|  | 3. I can make light of difficult situations | 11 | 16 | 0.69 | 0.67 | FALSE |
|  | 4. I easily laugh | 11 | 16 | 0.69 | 0.67 | FALSE |
|  | 5. I laugh often | 13 | 16 | 0.81 | 0.81 | TRUE |
|  | 6. It is easy for me to make other people laugh | 8 | 16 | 0.50 | 0.38 | FALSE |
|  | 7. I laugh with my nursing colleagues | 14 | 16 | 0.88 | 0.88 | TRUE |
|  | 8. I use humour in my interactions with patients | 13 | 16 | 0.81 | 0.81 | TRUE |
|  | 9. I make and share jokes with my colleagues | 10 | 16 | 0.63 | 0.57 | FALSE |
|  | 10. I can usually see the funny side of things | 15 | 16 | 0.94 | 0.94 | TRUE |
|  | 11. I find things to laugh about | 12 | 16 | 0.75 | 0.74 | FALSE |
|  | 12. I find humour in aspects of my work | 12 | 16 | 0.75 | 0.74 | FALSE |
|  | 13. I use humour to relieve tension | 14 | 16 | 0.88 | 0.88 | TRUE |
|  |  |  |  |  |  |  |
| ***Optimism*** | |  |  |  |  |  |
|  | 1. I like to focus on the positives rather than the negatives | 15 | 16 | 0.94 | 0.94 | TRUE |
|  | 2. During and after life's most stressful events I tend to look for creative solutions to the problem | 14 | 16 | 0.88 | 0.88 | TRUE |
|  | 3. There is an optimistic feeling among my nursing colleagues about the work we do | 15 | 16 | 0.94 | 0.94 | TRUE |
|  | 4. During and after life's most stressful events I tend to find an opportunity for growth | 15 | 16 | 0.94 | 0.94 | TRUE |
|  | 5. I find it easy to think of the positives when faced with difficulty | 15 | 16 | 0.94 | 0.94 | TRUE |
|  | 6. During and after life's most stressful events I tend to find strength in the meaning, purpose, or mission of my life | 14 | 16 | 0.88 | 0.88 | TRUE |
|  | 7. I often feel hopeful | 15 | 16 | 0.94 | 0.94 | TRUE |
|  | 8. I believe I can grow in positive ways by dealing with difficult situations | 16 | 16 | 1.00 | 1.00 | TRUE |
|  | 9. I am optimistic about the future of healthcare provision | 14 | 16 | 0.88 | 0.88 | TRUE |
|  | 10. During and after life's most stressful events I tend to see it as a challenge that will make me better | 11 | 16 | 0.69 | 0.67 | FALSE |
|  | 11. I have a positive outlook | 15 | 16 | 0.94 | 0.94 | TRUE |
|  | 12. I am optimistic about my work | 15 | 16 | 0.94 | 0.94 | TRUE |
|  | 13. I see challenges as an opportunity to grow | 16 | 16 | 1.00 | 1.00 | TRUE |
|  | 14. I feel happy often | 13 | 16 | 0.81 | 0.81 | TRUE |
|  | 15. There is an optimistic feeling among my multidisciplinary team colleagues about the work we do | 13 | 16 | 0.81 | 0.81 | TRUE |
|  | 16. I am optimistic about the future of the nursing profession | 10 | 16 | 0.63 | 0.57 | FALSE |
|  | 17. I pay more attention to the positive aspects of my profession | 11 | 16 | 0.69 | 0.67 | FALSE |
|  | 18. I feel good about my future | 14 | 16 | 0.88 | 0.88 | TRUE |
|  | 19. I am looking forward to my future | 14 | 16 | 0.88 | 0.88 | TRUE |
|  | 20. Generally I find things tend to work out for the best | 11 | 16 | 0.69 | 0.67 | FALSE |
|  | 21. During and after life's most stressful events I tend to practice ways to handle it better next time | 13 | 16 | 0.81 | 0.81 | TRUE |
|  | 22. My future feels promising | 12 | 16 | 0.75 | 0.74 | FALSE |
|  |  |  |  |  |  |  |
| ***Realistic - Professional & Personal*** | |  |  |  |  |  |
|  | 1. The demands of my job are realistic | 13 | 16 | 0.81 | 0.81 | TRUE |
|  | 2. I set achievable goals for myself | 13 | 16 | 0.81 | 0.81 | TRUE |
|  | 3. I am not asked to do anything outside my scope of practice | 14 | 16 | 0.88 | 0.88 | TRUE |
|  | 4. I have realistic plans for the future | 13 | 16 | 0.81 | 0.81 | TRUE |
|  | 5. I maintain a realistic perspective on what I can achieve | 14 | 16 | 0.88 | 0.88 | TRUE |
|  | 6. During and after life's most stressful events I tend to put things in perspective and realise I will have times of joy and times of sadness | 14 | 16 | 0.88 | 0.88 | TRUE |
|  | 7. It’s okay that not every day at work will be a good day | 15 | 16 | 0.94 | 0.94 | TRUE |
|  | 8. I am not asked to do anything that I have not been taught | 12 | 16 | 0.75 | 0.74 | FALSE |
|  | 9. I understand that my patients won’t always have positive outcomes | 16 | 16 | 1.00 | 1.00 | TRUE |
|  | 10. During and after life's most stressful events I tend to understand that bad things can happen to anyone not just me | 11 | 16 | 0.69 | 0.67 | FALSE |
|  | 11. During and after life's most stressful events I tend to be good at determining what situations are changeable and what situations are not | 14 | 16 | 0.88 | 0.88 | TRUE |
|  | 12. I am able to cope with the pressure of my work | 14 | 16 | 0.88 | 0.88 | TRUE |
|  |  |  |  |  |  |  |
| ***Workplace Conditions*** | |  |  |  |  |  |
|  | 1. I am sure that there is sufficient staffing to replace and support during a crisis or medical emergencies | 12 | 16 | 0.75 | 0.74 | FALSE |
|  | 2. Patient care in my hospital/organisation is of high quality | 16 | 16 | 1.00 | 1.00 | TRUE |
|  | 3. Patient care in my ward/department/setting is of high quality | 16 | 16 | 1.00 | 1.00 | TRUE |
|  | 4. Verbal and physical abuse is not tolerated in my workplace | 15 | 16 | 0.94 | 0.94 | TRUE |
|  | 5. I am able to take time off of work when I need it | 16 | 16 | 1.00 | 1.00 | TRUE |
|  | 6. Bullying is not tolerated in my workplace | 14 | 16 | 0.88 | 0.88 | TRUE |
|  | 7. I have a say in the conditions of my job | 16 | 16 | 1.00 | 1.00 | TRUE |
|  | 8. The patient care I am able to deliver is of high quality | 16 | 16 | 1.00 | 1.00 | TRUE |
|  | 9. My work is recognised in meaningful ways | 16 | 16 | 1.00 | 1.00 | TRUE |
|  | 10. I have adequate time to do my job | 16 | 16 | 1.00 | 1.00 | TRUE |
|  | 11. I have access to the equipment I need to perform well at work | 16 | 16 | 1.00 | 1.00 | TRUE |
|  | 12. I feel supported in my workplace | 16 | 16 | 1.00 | 1.00 | TRUE |
|  | 13. There are sufficient resources to do my job effectively | 16 | 16 | 1.00 | 1.00 | TRUE |
|  | 14. My workplace has adequate (enough) staffing | 14 | 16 | 0.88 | 0.88 | TRUE |
|  | 15. I am paid fairly for the work I do | 15 | 16 | 0.94 | 0.94 | TRUE |
|  | 16. My workplace is flexible when required | 16 | 16 | 1.00 | 1.00 | TRUE |
|  | 17. My workplace feels safe | 16 | 16 | 1.00 | 1.00 | TRUE |
|  | 18. My nursing team has an adequate skill mix | 15 | 16 | 0.94 | 0.94 | TRUE |
|  | 19. The multidisciplinary team has an adequate skill mix | 13 | 16 | 0.81 | 0.81 | TRUE |
|  | 20. The work I do is within the scope of my practice | 16 | 16 | 1.00 | 1.00 | TRUE |
|  |  |  |  |  |  |  |
| ***Organisational Philosophy*** | |  |  |  |  |  |
|  | 1. I see the organisation’s philosophy (or vision or mission statement) in action | 15 | 16 | 0.94 | 0.94 | TRUE |
|  | 2. The organisation’s philosophy (or vision or mission statement) is contemporary | 9 | 16 | 0.56 | 0.47 | FALSE |
|  | 3. The organisation where I work has a clear philosophy of nursing that pervades the patient care environment | 13 | 16 | 0.81 | 0.81 | TRUE |
|  | 4. The organisation I work for upholds the values it promotes | 15 | 16 | 0.94 | 0.94 | TRUE |
|  | 5. I feel like I am a good match for this organisation | 15 | 16 | 0.94 | 0.94 | TRUE |
|  | 6. My values align with the organisation’s philosophy (or vision or mission statement) | 14 | 16 | 0.88 | 0.88 | TRUE |
|  | 7. I feel personally valued by the organisation where I work | 16 | 16 | 1.00 | 1.00 | TRUE |
|  | 8. The organisation I work for values nurses | 15 | 16 | 0.94 | 0.94 | TRUE |
|  | 9. I fit with the culture of the organisation where I work | 15 | 16 | 0.94 | 0.94 | TRUE |
|  | 10. I know the organisation’s philosophy (or vision or mission statement) | 7 | 16 | 0.44 | 0.32 | FALSE |
|  | 11. I agree with the values promoted by my workplace | 14 | 16 | 0.88 | 0.88 | TRUE |
|  | 12. The organisation’s philosophy (or vision or mission statement) is relevant | 11 | 16 | 0.69 | 0.67 | FALSE |
|  |  |  |  |  |  |  |
| ***Management Performance*** | |  |  |  |  |  |
|  | 1. The Director of Nursing (or equivalent) is a “good” leader | 14 | 16 | 0.88 | 0.88 | TRUE |
|  | 2. My nurse manager empowers me in my work | 16 | 16 | 1.00 | 1.00 | TRUE |
|  | 3. High standards of nursing care are expected by the hospital/organisation executive team | 14 | 16 | 0.88 | 0.88 | TRUE |
|  | 4. My nurse manager encourages me to solve problems | 15 | 16 | 0.94 | 0.94 | TRUE |
|  | 5. I feel supported by my nurse manager | 16 | 16 | 1.00 | 1.00 | TRUE |
|  | 6. I have a nurse manager that is supportive of the nurses | 15 | 16 | 0.94 | 0.94 | TRUE |
|  | 7. My nurse manager inspires the team | 15 | 16 | 0.94 | 0.94 | TRUE |
|  | 8. My nurse manager discusses shared affairs with me | 6 | 16 | 0.38 | 0.29 | FALSE |
|  | 9. The hospital/organisation executive team listens and responds to employee concerns | 16 | 16 | 1.00 | 1.00 | TRUE |
|  | 10. Clinically based nurses are involved in the internal governance of the hospital/organisation (e. g. , practice and policy committees) | 14 | 16 | 0.88 | 0.88 | TRUE |
|  | 11. I feel valued by my nurse manager | 16 | 16 | 1.00 | 1.00 | TRUE |
|  | 12. We have a Director of Nursing (or equivalent) equal in power and authority to other top-level hospital/organisation executives | 10 | 16 | 0.63 | 0.57 | FALSE |
|  | 13. My nurse manager is a good leader | 15 | 16 | 0.94 | 0.94 | TRUE |
|  | 14. My nurse manager listens to me | 15 | 16 | 0.94 | 0.94 | TRUE |
|  | 15. The hospital/organisation executive team are “good” leaders | 13 | 16 | 0.81 | 0.81 | TRUE |
|  | 16. My nurse manager is approachable | 16 | 16 | 1.00 | 1.00 | TRUE |
|  | 17. The area I work has a nurse manager who backs up the nursing staff in decision making, even if the conflict is with a physician/doctor | 16 | 16 | 1.00 | 1.00 | TRUE |
|  | 18. Nurse managers use mistakes as learning opportunities, not criticism | 16 | 16 | 1.00 | 1.00 | TRUE |
|  | 19. My nurse manager communicates well with the team | 16 | 16 | 1.00 | 1.00 | TRUE |
|  | 20. Nursing executives consult with staff on daily problems and procedures | 11 | 16 | 0.69 | 0.67 | FALSE |
|  | 21. My nurse manager addresses my concerns | 16 | 16 | 1.00 | 1.00 | TRUE |
|  | 22. My nurse manager is understanding when I am having difficulties | 16 | 16 | 1.00 | 1.00 | TRUE |
|  |  |  |  |  |  |  |
| ***Team Factors*** | |  |  |  |  |  |
|  | 1. The culture of the area I work in is positive | 16 | 16 | 1.00 | 1.00 | TRUE |
|  | 2. My team supports me | 16 | 16 | 1.00 | 1.00 | TRUE |
|  | 3. The culture of the hospital/organisation is positive | 15 | 16 | 0.94 | 0.94 | TRUE |
|  | 4. I feel a sense of belonging with my team | 16 | 16 | 1.00 | 1.00 | TRUE |
|  | 5. I feel we are respected by our medical colleagues | 15 | 16 | 0.94 | 0.94 | TRUE |
|  | 6. Team work is supported and promoted in my workplace | 16 | 16 | 1.00 | 1.00 | TRUE |
|  | 7. There are sufficient nurses to meet patient care needs | 16 | 16 | 1.00 | 1.00 | TRUE |
|  | 8. My medical colleagues listen to concerns | 15 | 16 | 0.94 | 0.94 | TRUE |
|  | 9. When problems occur at work my team solves them together | 16 | 16 | 1.00 | 1.00 | TRUE |
|  | 10. The multidisciplinary team I work within has the skills needed to meet patient care needs | 15 | 16 | 0.94 | 0.94 | TRUE |
|  | 11. I work with a good team | 15 | 16 | 0.94 | 0.94 | TRUE |
|  | 12. I trust the people I work with | 16 | 16 | 1.00 | 1.00 | TRUE |
|  | 13. I feel respected by my fellow nursing colleagues | 16 | 16 | 1.00 | 1.00 | TRUE |
|  | 14. Professional collaboration across the multidisciplinary team is expected | 16 | 16 | 1.00 | 1.00 | TRUE |
|  | 15. I can communicate appropriately with the care team during a crisis or medical emergencies | 16 | 16 | 1.00 | 1.00 | TRUE |
|  | 16. I work closely with other members of the care team during a crisis or medical emergencies | 16 | 16 | 1.00 | 1.00 | TRUE |
